# Therapeutic Use of Mesenchymal Stem Cell-Derived Exosomes: From Basic Science to Clinics

**DOI:** 10.3390/pharmaceutics12050474

**Published:** 2020-05-22

**Authors:** Carl Randall Harrell, Nemanja Jovicic, Valentin Djonov, Vladislav Volarevic

**Affiliations:** 1 Regenerative Processing Plant, LLC, 34176 US Highway 19 N Palm Harbor, Palm Harbor, FL 34684, USA; dr.harrell@regenerativeplant.org; 2 Department of Histology and Embryology, Faculty of Medical Sciences, University of Kragujevac, Svetozara Markovica 69, 34000 Kragujevac, Serbia; nemanjajovicic.kg@gmail.com; 3 Institute of Anatomy, University of Bern, 2 Baltzerstrasse, 3012 Bern, Switzerland; valentin.djonov@ana.unibe.ch; 4 Department for Microbiology and Immunology, Center for Molecular Medicine and Stem Cell Research, Faculty of Medical Sciences, University of Kragujevac, 69 Svetozar Markovic Street, 34000 Kragujevac, Serbia; 5 Center of Excellence for the Acceleration of Harm Reduction (CoEHAR), Università di Catania, Via Santa Sofia 78, 95123 Catania, Italy

**Keywords:** mesenchymal stem cells, exosomes, inflammation, regeneration, therapy

## Abstract

Mesenchymal stem cells (MSC) are, due to their immunosuppressive and regenerative properties, used as new therapeutic agents in cell-based therapy of inflammatory and degenerative diseases. A large number of experimental and clinical studies revealed that most of MSC-mediated beneficial effects were attributed to the effects of MSC-sourced exosomes (MSC-Exos). MSC-Exos are nano-sized extracellular vesicles that contain MSC-derived bioactive molecules (messenger RNA (mRNA), microRNAs (miRNAs)), enzymes, cytokines, chemokines, and growth factors) that modulate phenotype, function and homing of immune cells, and regulate survival and proliferation of parenchymal cells. In this review article, we emphasized current knowledge about molecular and cellular mechanisms that were responsible for MSC-Exos-based beneficial effects in experimental models and clinical trials. Additionally, we elaborated on the challenges of conventional MSC-Exos administration and proposed the use of new bioengineering and cellular modification techniques which could enhance therapeutic effects of MSC-Exos in alleviation of inflammatory and degenerative diseases.

## 1. Introduction

Mesenchymal stem cells (MSCs) are self-renewable, adult stem cells that reside in almost all postnatal tissue and organs [1]. MSCs interact with parenchymal cells and promote repair and regeneration of injured tissues in juxtacrine and paracrine manner [1,2]. Damage associated molecular patterns and alarmins, released from injured cells, induce activation of MSCs which, in turn, prevent apoptosis of un-injured parenchymal cells and stimulate their survival and proliferation [2]. MSCs suppress effector functions of inflammatory neutrophils, monocytes, T lymphocytes, natural killer (NK), and natural killer T (NKT) cells and promote generation and expansion of immunosuppressive T regulatory cells (Tregs) leading to the alleviation of on-going inflammation [3]. Additionally, MSCs induce neo-angiogenesis and promote homing of alternatively activated macrophages and tolerogenic dendritic cells (DCs) into the inflamed tissues where these immunoregulatory cells enhance endogenous healing process [4]. Therefore, due to their immunosuppressive and regenerative properties, MSCs have been considered as potentially new therapeutic agents in the treatment of inflammatory and degenerative diseases.

Although MSC-dependent neo-vascularization, increased viability of parenchymal cells and immunosuppression significantly contributed to the enhanced repair and regeneration of injured and inflamed tissues, several lines of evidence indicated potential unwanted effects of MSC-based cell therapy [5]. Results obtained in animal models suggested that engrafted MSCs, in response to the growth factors produced within the local microenvironment, could give rise to unwanted cells, mainly osteocytes and chondrocytes [6,7]. Due to the low surface expression of major histocompatibility class (MHC) I and II antigens, MSCs were considered as “hypoimmunogenic” or “immune privileged” cells [8]. However, transplantation of allogeneic MSCs induced activation of immune responses in several MHC mismatched recipients [8]. Therefore, transplantation of MSCs still raises safety concerns in clinical settings [5].

The vast majority of MSC-based beneficial effects were relied on the activity of MSC-derived immunosuppressive, angiomodulatory, and trophic factors [9]. Additionally, side effects related to the clinical application of MSCs were not observed in animals and patients that were treated with MSC-derived secretome [5]. Therefore, therapeutic use of MSC-sourced secretome is currently considered as a potential substitution for MSC-cell based therapy [9].

MSC-sourced secretome contains MSC-derived bioactive molecules which are either dissolved in medium or enveloped within encapsulated extracellular vesicles (MSC-EVs): apoptotic bodies, microvesicles, and exosomes (Exos), distinguishable by their size [9]. While apoptotic bodies represent the biggest EVs (>1000 nm), MSC-derived microvesicles (100–1000 nm) and Exos (30–200 nm) have overlapping size ranges (100–200 nm) and methods currently used to separate these two sub-populations of EVs had varying degrees of success. Therefore, when separation could not be completely ascertained, these two MSC-sourced encapsulated products were collectively designated as MSC-derived EVs [9]. On the contrary, when MSC-Exos, as the smallest MSC-EVs originated via the inward budding of the late endosome membranes, were successfully isolated and characterized (mostly by the expression of tetraspanin proteins CD9, CD63, and CD81), therapeutic effects of MSC-sourced secretome was attributed to the activity of MSC-Exo-delivered factors [9].

Due to their nano-sized dimension, MSC–Exos, distributed via biological fluids, easily penetrate through the tissues and reach the target cells (even distant one), enabling both paracrine and endocrine effects [10]. MSC-Exos have lipid bilayers enriched with integrins and ligands for cell surface receptors [11]. Therefore, MSC-Exos deliver their content to the cytosol of target cells either through the direct fusion with the plasma membrane or through the ligand-based activation of membrane-bound receptors which results in activation of cytoskeletal proteins leading to the creation of internalized vacuole and internalization of MSC-Exo-sourced content [9].

**Figure 1:**
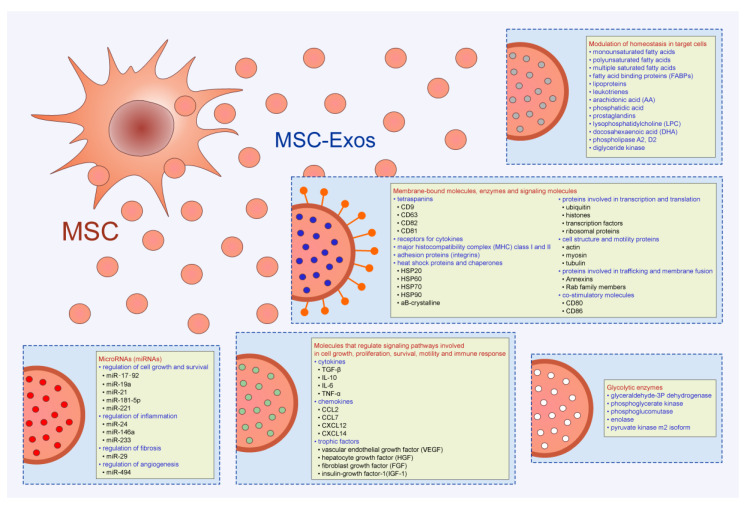
Composition of Mesenchymal stem cells- exosomes (MSC-Exos) and their role in MSC-Exo-mediated biological effects. MSC-Exos contain a large number of biologically active molecules (lipids, proteins (enzymes, cytokines, immunoregulatory proteins, growth factors, and miRNAs) which are responsible for MSC-Exo-based therapeutic effects.

Therapeutic potential of MSC-Exos is relied on the effects of MSC-sourced bioactive molecules (lipids, proteins (enzymes, cytokines, chemokines, immunoregulatory proteins, and trophic and growth factors), microRNAs (miRNAs),) which efficiently modulate immune response and promote tissue repair and regeneration (Figure 1) [10]. In line with these findings, a large number of experimental and clinical studies investigated signaling pathways, molecular and cellular mechanisms responsible for the beneficial effects of MSC-Exos [9]. In this review article, we emphasized current knowledge and future perspectives related to the therapeutic use of MSC-Exos in the treatment of inflammatory and degenerative diseases.

## 2. Components of MSC-Exos and Their Role in MSC-Exo-Mediated Biological Effects

MSC-Exos contain a broad spectrum of monounsaturated, polyunsaturated, and multiple saturated fatty acids, fatty acid binding proteins (FABP), and lipoproteins [12]. MSC-Exo-derived leukotrienes, arachidonic acid (AA), phosphatidic acid, prostaglandins lysophosphatidylcholine (LPC), docosahexaenoic acid (DHA), and enzymes that regulate lipid metabolism (phospholipase A2, D2, and diglyceride kinase) play important role in the MSC-Exo-based modulation of homeostasis in target tissues. Phosphatidylserine and LPC, expressed on MSC-Exos, are recognized by recipient cells through TIM (T cell/transmembrane, immunoglobulin, and mucin) and G protein coupled receptors. Once they enter in the target cell, MSC-Exo-sourced FABP and AA form FABP–AA complex which enters in the nucleus by binding to the nuclear receptor Peroxisome proliferator-activated receptor gamma (PPARγ). Within the nucleus of recipient cell, MSC-Exo-derived FABP–AA complex regulates expression of genes involved in cell growth, apoptosis and metabolism, affecting survival of target cell [12].

More than 2000 proteins, including membrane-bound molecules, enzymes, and signaling molecules, are identified in MSC-Exos [12,13]. Tetraspanins (CD9, CD63, CD82, and CD81), receptors for cytokines, proteins involved in antigen-presentation (MHC class I and II proteins and co-stimulatory molecules (CD80, CD86)) and adhesion proteins (integrins)) are expressed on the membrane of MSC-Exos [14]. Heat shock proteins and chaperones (HSP20, HSP60, HSP70, HSP90, and aB-crystalline), transcription and translation-related proteins (ubiquitin, histones, transcription factors, and ribosomal proteins), cell structure and motility proteins (actin, myosin, and tubulin) and proteins involved in trafficking and membrane fusion (Annexins and Rab family members) are present in MSC-Exos [12]. Additionally, MSC-Exos contain large number of cytokines (transforming growth factor beta (TGF-β), interleukin (IL)-10, IL-6, tumor necrosis factor alpha (TNF-α), etc.), chemokines (CCL2, CCL7, CXCL12, CXCL14, etc.) and trophic factors (vascular endothelial growth factor (VEGF), hepatocyte growth factor, fibroblast growth factor (FGF), and insulin-growth factor-1) that regulate signaling pathways involved in cell growth, proliferation, survival, motility, and immune response [14]. Proteomic studies revealed that MSC-Exos contain five glycolytic enzymes (glyceraldehyde-3Pdehydrogenase, phosphoglycerate kinase, phosphoglucomutase, enolase, and pyruvate kinase m2 isoform) and are, therefore, able to generate glycolytic adenosine triphosphate (ATP). Interestingly, ATP-generating potential of MSC-Exos could be enhanced by culture conditioning [12]. MSCs pretreated with oligomycin (which inhibits mitochondrial ATPase), produce MSC-Exos with enhanced activity of glycolytic enzymes. Accordingly, Exos, obtained from oligomycin-treated MSCs, significantly increase ATP generation after injection in ischemic microenvironment. Hypoxic culture conditions significantly up-regulated expression of angiogenesis-related factors (platelet-derived growth factor, epidermal growth factor, FGF) in MSC-Exos, enhancing their angio-modulatory properties [12].

In addition to lipids and proteins, MSC-Exos are also enriched in miRNAs, small, noncoding RNA molecules, which regulate cell growth and survival (miR-17-92, miR-19a, miR-21, miR-181-5p, miR-221), inflammation (miR-24, miR-146a, miR-233), fibrosis (miR-29), and angiogenesis (miR-494) [13].

## 3. Molecular and Cellular Mechanisms Responsible for Immunomodulatory Effects of MSC-Exos

A large number of experimental studies demonstrated that MSC-Exos efficiently inhibited effector function of inflammatory M1 macrophages, alleviated antigen presenting properties of DCs, suppressed generation of inflammatory CD4+ Th1 and Th17 lymphocytes and induced expansion of regulatory and immunosuppressive Tregs, tolerogenic DCs and alternatively activated macrophages, contributing to the attenuation of on-going inflammation [14].

### 3.1. MSC-Exo-Based Inhibition of Inflammatory Macrophages

Significantly reduced expression of inducible nitric oxide synthase (iNOS) and inflammatory cytokines (TNF-α, IL-1β, and IL-6) were observed in MSC-Exo-treated macrophages [15,16]. As demonstrated by Wu and colleagues, MSC-Exos delivered microRNA-146a (miR-146a) in the cytosol of macrophages and, in miR-146a-dependent manner inhibited TNF receptor-associated factor 6 (TRAF6) and IL-1 receptor-associated kinase 1 (IRAK1), resulting in the down-regulated phosphorylation of NF-κB p65 [15]. Suppression of NF-κB signaling pathway alleviated expression of iNOS, TNF-α, IL-1β, and IL-6 genes and inhibited generation of inflammatory M1 phenotype in MSC-Exo-treated macrophages [15]. Additionally, MSC-Exos suppressed production of IL-7 and prevented IL-7:IL-7R-dependent migration of circulating monocytes in inflamed tissues [16]. In line with these findings, Mao and coworkers demonstrated that 12h after intravenous injection, MSC-Exos accumulated in the inflamed colons of dextran sodium sulfate (DSS)-treated mice and efficiently suppressed effector functions of inflammatory, M1 colonic macrophages leading to the alleviation of DSS-induced colitis [16].

Similarly, MSC-Exos suppressed production of inflammatory cytokines (TNF-α, IL-1β, and IL-6) in liver macrophages (Kupffer cells) and reduced their cross-talk with pro-fibrotic hepatic stellate cells (HSCs) [17]. By preventing generation of inflammatory phenotype in Kupffer cells and by suppressing expression of pro-fibrotic genes (collagen I, vimentin, alpha-smooth muscle actin (α-SMA), and fibronectin) in HSCs, MSC-Exos significantly alleviated liver fibrosis in mice [17]. Qu and colleagues engineered miR-181-5p-overexpressing MSCs-Exos which induced enhanced expression of autophagy-related Beclin-1 and suppressed expression of anti-apoptotic Bcl-2 in HSCs, resulting in their increased apoptosis loss [17]. Significantly reduced number of M1 macrophages and HSCs in the injured livers of MSCsmiR-181-5p-Exos-treated mice led to the attenuation of chronic liver inflammation and fibrosis [17].

### 3.2. Effects of Aging on MSC-Exo-Dependent Generation of Immunosuppressive Phenotype in Macrophages

In addition to the suppression of M1 macrophages, MSC-Exos induced generation of immunosuppressive M2 phenotype in macrophages by enhancing expression of Arginase-1 [18]. Significantly higher number of IL-10 and TGF-β-producing, alternatively activated M2 alveolar macrophages were found in the lungs of MSC-Exo-treated mice leading to the alleviation of ischemia/reperfusion (I/R) lung injury [18]. Importantly, aging negatively affected MSC-Exos-based modulation of macrophage function [19]. As recently demonstrated by Huang and coworkers, Aging MSC-Exos (obtained from 72-years old donor) expressed different levels of immunoregulatory miRNAs compared to the young MSC-Exos (obtained from 25-years old donor) and were not able to induce generation of immunosuppressive phenotype in macrophages in the same manner as young MSC-Exos [19]. Aging MSC-Exos did not manage to induce expression of Arginase 1 and failed to optimally suppress production of nitric oxide (NO), TNF-α, IL-1β, and IL-6 in macrophages [19]. The difference in the content of miR-223-5p, miR-127-3p, and miR-125b-5p, which regulate macrophage polarization, was responsible for impaired capacity of aging MSC-Exos in modulation of macrophage function. In MSC-Exos, expression of M2-inducing miR-223-5p decreased, while expression of M1-inducing miR-127-3p and miR-125b-5p increased with aging [19], suggesting that only MSC-Exos obtained from younger donors should be used for the attenuation of macrophage-driven inflammatory diseases. It is well known that aging impairs capacity of MSCs to produce immunomodulatory miRNAs [20]. The content of miRNAs that regulated inflammation, oxidative stress and mitochondrial function (miR-132, miR-146a, miR-155, miR-294, and miR-872-3p) in MSC-Exos decreased with the age of MSC donors [20]. Additionally, aging altered expression of MSC-sourced miRNAs that regulate differentiation and tissue regeneration [21]. Wang and colleagues revealed that, due to the down-regulated expression of miR-133b-3p and miR-294, MSC-Exos from older rats were weaker in inhibiting epithelial–mesenchymal transition than younger rats [21]. Fafian-Labora and coworkers compared expression of miRNAs in MSCs-Exos obtained from the newborn (0 days old), infant (7 days old), young (14 days old), pre-pubertal (35–38 days old), pubertal (45 days old), and adult (108 days old) rats and concluded that alteration of miRNA levels in MSC-Exos might serve as a marker of aging [22]. They observed that levels of miR-146a, miR-155, and miR-132 significantly decreased with aging. The lowest expression of miR-335 was noticed in Exos derived from young MSC donors, while the high expression of miR-21 was a hallmark of MSC-Exos obtained from the pre-pubertal donors [22].

### 3.3. MSC-Exo-Mediated Generation of Immunosuppressive Phenotype in Microglial Cells

In a similar manner as it was observed in the colon, liver, and alveolar macrophages, MSC-Exos induced generation of anti-inflammatory M2 phenotype in microglial cells, as well [23]. Capacity for production of immunosuppressive cytokines (IL-10 and TGF-β) significantly increased while expression of inflammatory cytokines (TNF-α and IL-1β) decreased in MSC-Exo-treated BV2 murine microglia cells [23]. Results obtained in several experimental studies suggested that MSC-Exos-mediated generation of anti-inflammatory M2 phenotype in microglia cells crucially contributed to the attenuation of Alzheimer’s disease (AD) and multiple sclerosis (MS) [23,24]. Systemically infused MSC-Exos induced expansion of Arginase-1 and Mannose receptor C type 1 (MRC1)-expressing M2 microglia cells and reduced accumulation of the amyloid-β peptide (Aβ) in the brains of AβPP/PS1 transgenic mice, used as a murine model of AD [23]. Through the secretion of Aβ-degrading enzymes (Neprilysin (NEP) and Insulin-degrading enzyme (IDE)), M2 microglial cells prevented accumulation of Aβ in the brains, while in IL-10 and TGF-β-dependent manner attenuated on-going inflammation, resulting in the improvement of cognitive function in experimental animals [23]. In similar manner, MSC-Exos altered cytokine profile of Iba-1-expressing microglial cells in the brains of Theiler’s murine encephalomyelitis virus (TMEV)-infected mice, used as a model of MS [24]. Remarkably improved motor function, observed in TMEV+MSC-Exo-treated animals, corresponded to the alleviated production of inflammatory cytokines (TNF-α, IL-1-β, IL-18, IL-6, and IL-12) in MSC-Exo-treated microglial cells [24]. A significantly decreased concentration of Th1 cell-sourced IFN-γ and Th17 cell-derived IL-17 in the brains of MSC-Exo-treated animals, suggested that MSC-Exos, in addition to microglial cells, also suppressed inflammatory properties of brain-infiltrating CD4+T cells [24].

### 3.4. Effects of MSC-Exos on Antigen-Presenting Properties of DCs

**Figure 2:**
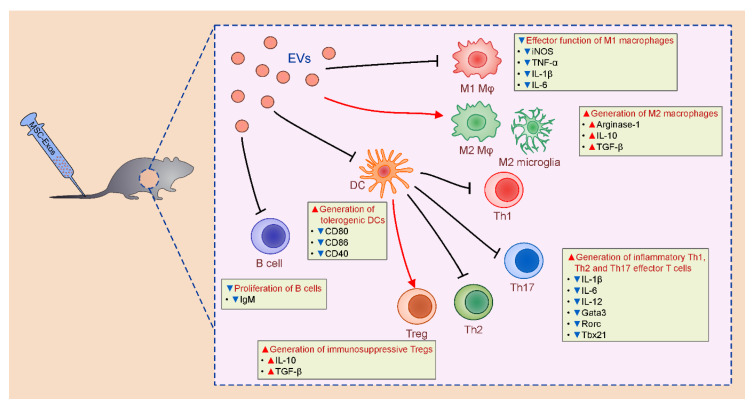
Immunomodulatory effects of MSC-Exos. MSC-Exos modulated effector function of dendritic cells (DCs), macrophages, T and B lymphocytes. MSC-Exos attenuated antigen-presenting properties of DCs, inhibited proliferation of B lymphocytes, suppressed generation of inflammatory M1 macrophages, Th1, Th2, and Th17 cells and induced enhanced expansion of immunosuppressive Tregs, tolerogenic DCs, and alternatively activated, M2 macrophages in experimental animals.

MSC-Exos-mediated suppression of Th1 and Th17 cells was mainly a consequence of their effect on antigen-presenting properties of DCs (Figure 2) [14]. Shahir and colleagues recently demonstrated that MSC-Exos alleviated T cell-driven inflammatory diseases by inducing generation of immunosuppressive, tolerogenic phenotype in murine DCs [25]. Significantly reduced expression of co-stimulatory molecules (CD80, CD86, and CD40) were observed on the membranes of MSC-Exo-treated DCs, suggesting their reduced capacity for activation of naïve T cells. In line with these findings, remarkably lower proliferation of T lymphocytes was observed in the presence of DCs which were previously primed by MSC-Exos [25]. Additionally, significantly higher production of immunosuppressive cytokines (IL-10 and TGF-β) and notably reduced release of inflammatory IL-6 were observed in DCs that were cultured with MSC-Exos, suggesting that MSC-Exos enhanced immunosuppressive properties of DCs [25].

MSC-Exo-primed DCs had reduced capacity for generation of inflammatory Th1, Th2, and Th17 effector T cells [26,27]. MSC-Exo-mediated modulation of antigen-presenting properties of lung DCs resulted in attenuated activation of CD4+Th2 cells and consequently led to the alleviation of Th2-driven immune response towards Aspergillus protease antigen [26]. In the inflamed kidneys, MSC-Exo-sourced miR-21 inhibited maturation of renal DCs by suppressing activation of NF-κB signaling pathway [27]. MSC-Exo-primed immature DCs were not able to optimally activate renal-infiltrating inflammatory Th1 and Th17 cells. Therefore, significantly alleviated Th1 and Th17 cell-driven renal inflammation and attenuated acute kidney injury were noticed in animals that were treated with miR-21-expressing MSC-Exos [27]. Similarly, MSC-Exos significantly reduced expression of co-stimulatory molecules and MHC class II proteins on retinal DCs and down-regulated their capacity for production of Th1 and Th17-related cytokines (IL-1β, IL-6, and IL-12) leading to the alleviation of Th1 and Th17 cell-driven experimental autoimmune uveoretinitis (EAU) [28].

Fathollahi and coworkers analyzed expression of genes that regulate MSC-Exo-based suppression of effector CD4+Th1, Th2, and Th17 cells [29]. Effects of MSC-Exos were analyzed in MSC-Exo-treated CD4+T lymphocytes that were isolated from experimental autoimmune encephalomyelitis (EAE) mice and in vitro re-stimulated by EAE-inducing peptide (MOG35-55). MSC-Exos treatment significantly down-regulated expression of Tbx21 and Gata3 genes which acted as the master regulators for Th1 and Th2 immune responses. Similarly, generation of inflammatory Th17 subset of CD4+ lymphocytes was inhibited by MSC-Exos, as evidenced by significantly reduced expression of Rorc gene that regulated differentiation of naïve T cells in effector Th17 cells [29]. MSC-Exo-mediated suppression of Th1 and Th17 cells also showed beneficial effects in alleviation of streptozotocin-induced model of type-1 diabetes mellitus (STZ-T1DM) in which these inflammatory CD4+T cells play pathogenic role [30].

### 3.5. MSC-Exo-Dependent Modulation of T Cells

In addition to the suppression of Th1 and Th17 cell-driven inflammation, MSC-Exo-primed DCs had increased capacity to induce generation of CD4+FoxP3-expressing immunosuppressive Tregs [31]. MSC-Exo-mediated, DC-dependent expansion of Tregs was crucially important for the alleviation of graft-versus-host disease (GVHD) and collagen-induced arthritis in mice [31,32]. Interestingly, MSC-Exos more efficiently suppressed proliferation of inflammatory Th1 and Th17 cells and induced expansion of immunosuppressive Tregs than their parental MSC. Although the main reason for this differential activity is still unknown, several research groups indicated important role of TGF-β signaling for this phenomenon [32]. Alvarez and colleagues [33] and Crain and coworkers [34] recently revealed that MSC-Exos mainly suppressed proliferation of activated CD4+ T cells in TGF-β dependent manner. TGF-β was expressed on the membranes of MSC-Exos in the complex with betaglycan. After entering in the cytosol of T cells, MSC-Exo-sourced TGF-β suppressed activation of Jak-Stat signaling pathway and caused the G1 cell cycle arrest in inflammatory CD4+T cells, resulting in their reduced expansion [34,35]. Use of TGF-βRI antagonist or neutralizing antibodies to TGF-β, completely diminished immunosuppressive properties of MSC-Exo against activated CD4+T cells, confirming crucially important role of TGF-β for MSC-Exo-based inhibition of CD4+T cell proliferation [34]. On the contrary, simultaneous, DC-dependent, activation of T cell receptor (TCR) and TGF-β receptor on naïve CD4+CD25- T cells triggers IL-2 and Foxp3 expression, resulting in the generation of FoxP3-expressing Tregs [36]. Passive transfer of TGF-β-generated Tregs efficiently inhibited innate inflammatory response in pancreatic islets and enhance survival of diabetic mice, confirming crucially important role of MSC-derived TGF-β for generation of regulatory phenotype in CD4+T cells [14,36].

Zhang and colleagues recently revealed that in addition to TGF-β, indoleamine-2,3-dioxygenase (IDO) also played important role in MSC-Exo-mediated enhanced generation of Tregs [37]. IDO, produced either by MSCs or MSC-primed DCs, degrades tryptophan to kynurenine and other metabolites (quinolinic acid and 3-hydroxy-anthranillic acid) [38]. During TCR-mediated activation of resting Tregs, simultaneous activation of protein kinase B (PKB/Akt) and mammalian target of rapamycin (mTOR) pathways induce reprogramming of Tregs into a pro-inflammatory Th17 phenotype [39]. Increased IDO activity significantly attenuates tryptophan levels in the local microenvironment which activates stress-response pathways in Tregs, including activation of general control nonderepressible 2 (GCN2) kinase. GCN2, in turn, inhibits Akt/mTOR2 signaling pathways and prevents transdifferentiation of Tregs in Th17 cells [39]. Accordingly, MSC-Exos in IDO/Kynurenine dependent manner maintain anti-inflammatory phenotype of Tregs and support TGF-β-induced expansion of these immunosuppressive cells in inflamed tissues [14].

MSC-Exo-dependent expansion of Tregs had crucially important role in alleviation of streptozotocin-induced type-1 diabetes mellitus (STZ-T1DM) in mice [30]. MSC-Exos induced expansion of immunosuppressive, IL-10 and TGF-β-producing Tregs that resulted in elevated serum levels of anti-inflammatory IL-10 and TGF-β and, consequently, led to the alleviation of inflammation in pancreatic islets. Additionally, administration of MSC-Exos significantly reduced presence of Th1 and Th17 cells in pancreatic islets of STZ-treated mice that was followed by decreased serum levels of inflammatory cytokines (IFN-γ and IL-17) [30]. Body weight, blood glucose levels, and survival were maintained stable in MSC-Exo-treated T1DM mice while significant increase in the total number of un-injured islets were observed in MSC-Exo-treated T1DM mice in comparison to MSC-Exo-untreated T1DM animals [30]. By using asthmatic mice, Du and colleagues demonstrated that MSC-Exos efficiently alleviated chronic airway inflammation by promoting expansion of TGF-β and IL-10-producing Tregs in the lungs [40]. Similarly, significantly lower number of inflammatory Th1 and Th17 cells and remarkably higher presence of IL-10-producing FoxP3-expressing Tregs were observed in the lungs of cigarette smoke (CS)-exposed mice that received Exos obtained from amniotic fluid-derived MSCs (Exo-dMAPPS) [41]. Exo-dMAPPS-based therapy significantly improved respiratory function, down-regulated serum levels of inflammatory cytokines (TNF-α, IL-1β, IL-12, and IFN-γ), increased serum concentration of immunosuppressive IL-10 and attenuated chronic airway inflammation in CS-exposed mice [41].

### 3.6. Effects of MSC-Exos on Phenotype and Function of B Cells

As recently revealed by Khare and colleagues [42], MSC-Exos inhibited proliferation of B lymphocytes and alleviated their capacity for IgM production. MSC-Exos affected expression of 186 genes in activated B lymphocytes. Most of them are involved in maturation, trafficking, antigen-presenting and effector function of B lymphocytes. Through the delivery of miRNAs, MSC-Exos down-regulated expression of Joining Chain Of Multimeric IgA And IgM (JCHAIN), Prostaglandin-Endoperoxide Synthase 2 (PTGS2), POU Class 2 Homeobox Associating Factor 1 (POU2AF1), TNF Receptor Superfamily Member 13B (TNFRSF13B), and Lymphotoxin Alpha (LTA) genes that resulted in significantly reduced production of IgM in activated B lymphocytes. Interestingly, MSC-Exos increased expression of CXCL8 (IL-8) in B lymphocytes. IL-8 acts as a chemo-attractant for activated, follicular CD4+T cells enabling their interaction with antigen-presenting B cells [42]. Additionally, B cell-derived IL-8, in autocrine and paracrine manner, increased expression of MHC class II molecules, enhancing capacity of B cells for the presentation of protein antigens to the activated CD4+ T cells [42]. It is still unclear whether MSC-Exo-induced increased expression of IL-8 gene in B cells could enhance their capacity for generation of immunosuppressive phenotype in CD4+T cells and this hypothesis should be confirmed in future experimental studies.

In addition to their effect on phenotype and function of immune cells (Table 1), MSC-Exos also modulated migratory properties of inflammatory cells [43]. As evidenced by Bai and colleagues, MSC-Exos significantly alleviated EAU in rats by suppressing CCL2 and CCL21-dependent homing of circulating leukocytes in the inflamed eyes [43]. A remarkably lower number of inflammatory M1 macrophages and Th1 and Th17 lymphocytes were observed in injured retinas of MSC-Exo-treated rats as a consequence of MSC-Exo-mediated inhibition of CCL2 and CCL21 production [43].

## 4. Signaling Pathways Responsible for MSC-Exo-Dependent Enhanced Survival and Regeneration of Injured Parenchymal Cells

**Figure 3:**
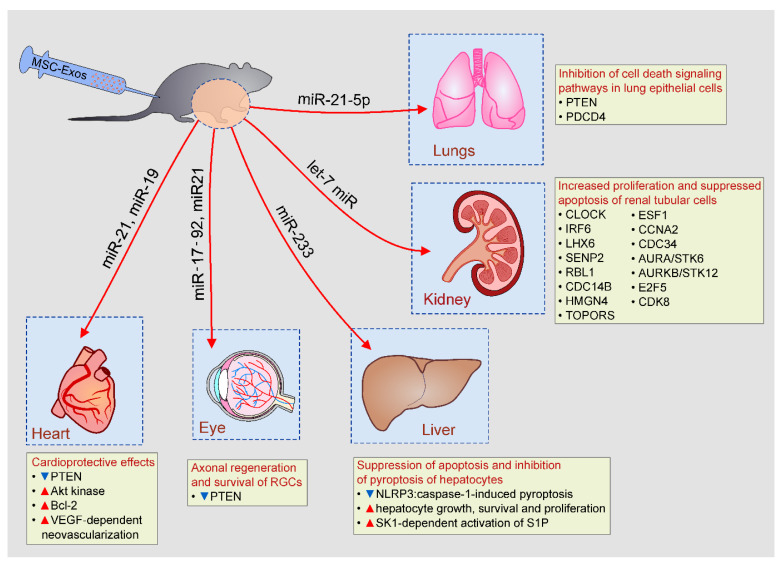
Beneficial effects of MSC-Exos in tissue repair and regeneration. MSC-Exos, in miR-19, miR-21, let-7c miR, miR-21-5p, miR-17-92, and miR-233-dependent manner, prevented apoptosis of lung and renal tubular epithelial cells, increased survival of cardiomyocytes in ischemic hearts, enhanced axonal regeneration and promoted hepatocyte growth and proliferation.

Through the delivery of miRNAs which interfere with cell death signaling pathways, MSC-Exos prevent apoptosis and promote survival of un-damaged parenchymal cells during the progression of tissue injury and inflammation (Figure 3) [13,14].

### 4.1. MSC-Exo-Based Protection of Lung Epithelial Cells, Renal Tubular Cells, and Hepatocytes

Li and coworkers emphasized important role of miR-21-5p for the MSC-Exo-dependent inhibition of cell death signaling pathways in lung epithelial cells (LECs) [18]. Pro-apoptotic proteins Phosphatase and tensin homolog (PTEN) and Programmed cell death protein 4 (PDCD4) were the main intracellular targets of miR-21-5p in MSC-Exo-based protection of LECs [44]. Pre-treatment of MSCs with miR-21-5p antagomir significantly abrogated capacity of MSC-Exos to suppress PTEN and PDCD4-induced apoptosis of LECs and completely diminished MSC-Exo-mediated therapeutic effects in alleviation of I/R-induced lung injury [18,44].

As suggested by Bruno and colleagues [45,46], mRNAs which regulate transcription (CLOCK, IRF6, and LHX6), cell cycle regulation (SENP2, RBL1, and CDC14B) and DNA/RNA repair (HMGN4, TOPORS, and ESF1) were mainly responsible for increased proliferation and suppressed apoptosis of renal tubular cells in cisplatin-injured kidneys of MSC-Exo-treated animals. Due to their capacity to modulate expression of apoptosis-related genes (CCNA2, CDC34, AURA/STK6, AURKB/STK12, E2F5, and CDK8), members of the let-7 miR family were also considered important for anti-apoptotic properties of MSC-Exos [47]. Importantly, MSC-Exo-based renoprotection was completely diminished by RNase pretreatment [48], confirming the hypothesis that beneficial effects of MSCs-Exos were mainly relied on the activity of MSC-Exo-delivered mRNA.

Intravenous injection of MSC-Exos prevented hepatocyte cell death in animal models of acute liver failure and autoimmune hepatitis [49,50]. Hepatoprotective effects of MSC-Exos were relied on the suppression of caspase-3-driven apoptosis and on inhibition of caspase-1-induced pyroptosis of hepatocytes [49,50]. MSC-Exos-dependent delivery of miR-233 induced degradation of NLRP3 mRNA in hepatocytes and suppressed NLRP3:caspase-1-induced pyroptosis [50]. Additionally, MSC-Exos increased hepatocyte number by inducing sphingosine kinase (SK1)-dependent activation of sphingosine-1-phosphate (S1P) which promoted hepatocyte growth, survival and proliferation [51]. Inhibition of either SK1 or S1P completely abrogated MSC-Exos-dependent expansion of hepatocytes, confirming crucially important role of SK1:S1P signaling pathway for hepatoproliferative effects of MSC-Exos [52].

### 4.2. MSC-Exo-Dependent Modulation of Apoptosis and Autophagy in Injured Parenchymal Cells

Several lines of evidence suggested that MSC-Exo-mediated activation of autophagy protected against apoptotic cell death [53]. MSC-Exo-dependent delivery of trophic factor 14-3-3ζ and consequent activation of autophagy associated protein ATG-16L inhibited apoptosis in cisplatin-injured proximal tubular epithelial cells [54]. Huang and colleagues revealed that MSC-Exos, through the delivery of pigment epithelium-derived factor (PEDF), increased expression of autophagy related protein LC3 and suppressed caspase-3-driven apoptosis in neurons, significantly reducing I/R-induced brain injury [55]. These MSC-Exo-mediated beneficial effects were completely abrogated by autophagy inhibitor, 3-methyladenine, confirming crucially important role of autophagy induction for anti-apoptotic effects of MSC-Exos [55,56]. In addition to their anti-apoptotic properties, MSC-Exos were also able to promote neuritogenesis [57]. Mead and Tomarev analyzed the effects of MSC-Exos on retinal ganglion cells (RGCs) and demonstrated that MSC-Exos, through the delivery of miR-17-92 and miR21, which down-regulated expression of PTEN (well-known suppressor of RGC axonal growth), promoted axonal regeneration, and survival of RGCs [57].

In line with these findings are results obtained by Wang and coworkers who demonstrated that miR-21 and miR-19-dependent inhibition of PTEN-driven apoptosis was crucially responsible for cardioprotective effects of MSC-Exos in animal model of acute myocardial infarction [58]. Through the delivery of miR-19, MSC-Exos down-regulated activation of PTEN and induced phosphorylation and activation of Akt kinase which, in turn, up-regulated anti-apoptotic Bcl-2 protein and reduced apoptotic loss of cardiomyocytes [58]. Additionally, MSC-Exos, in miR-21-dependent manner, improved cardiac function by inducing neoangiogenesis in ischemic hearts through the enhanced expression of VEGF [58]. Similar findings were reported by Shiue and colleagues who demonstrated that VEGF-dependent neovascularization was, at least partially, responsible for improved functional recovery from nerve ligation-induced injury in MSC-Exo-treated rats [59].

## 5. Clinical Use of MSC-Exos

Experimental studies indicated beneficial effects of MSC-Exos in the treatment of inflammatory and degenerative diseases and suggested their superiority to cell-based therapy in terms of safety. However, therapeutic potential of MSC-Exos had been explored in only several on-going or already conducted clinical trials. A relatively small number of MSC-Exo-related clinical studies could be explained by the fact that the optimal culture conditions and protocols for isolation, storage, and application of MSC-Exos are not defined yet [60]. Additionally, critical technological considerations related to the route and dose of MSC-Exos administration still need to be addressed [60]. Experimental findings indicated that route of administration and treatment schedule significantly affected therapeutic dose of MSC-Exos. Neuroprotection elicited by local, intravitreal injection of 3 × 10^9^ MSC-Exos was achieved by five times higher dose of intravenously injected MSC-Exos (15 × 10^9^ MSC-Exos). MSC-Exos reside in the eye up to 30 days and, accordingly, neurotrophic effects, elicited by single intravitreal injection of MSC-Exos, were completely diminished one month after application. On the contrary, permanent MSC-Exo-based neuroprotection was achieved by multiple, weekly injections of MSC-Exos, suggesting that repetitive or sustained delivery of MSC-Exos significantly enhanced their bioavailability and efficacy [61]. Therefore, the optimal therapeutic dose of MSC-Exos should be determined for each clinical condition and has to be based on the treatment schedule, route of administration and longevity of MSC-Exos in the target tissue.

The therapeutic efficacy of MSC-Exos in attenuation of chronic renal inflammation and MSC-Exo-mediated improvement of kidney function have been confirmed in the clinical study recently published by Nassar and colleagues [62]. MSC-Exos efficiently alleviated chronic kidney disease (grade III-IV) in 20 patients that received two doses (1 week apart) of MSC-Exos (100 μg/kg/dose). The first dose was injected intravenously and the second dose was administered via renal artery [62]. MSC-Exos alleviated TNF-α-driven inflammation and induced TGF-β and IL-10-dependent anti-inflammatory response that significantly improved estimated glomerular filtration rate (eGFR) and urinary albumin creatinine ratio. Importantly, any significant adverse events related to the administration of MSC-Exos were not reported during the one year of follow-up [62].

These promising results provided a rationale for the clinical use of MSC-Exos in a wide spectrum of diseases. Therapeutic potential of MSC-Exos in the treatment of acute ischemic stroke has been investigated in the clinical trial conducted at Isfahan University of Medical Sciences (NCT03384433). MSC-Exos were engineered to expressed miR-124 which already showed beneficial effects in animal model of ischemic stroke [63]. A single dose of miR-124-expressing MSC-Exos (200 mg of total protein) were injected into the ischemic area via stereotactic guidance. Incidence of treatment emergent adverse events (stroke recurrences, brain edema, and seizures) and degree of disability were monitored during the one year of follow-up. The study was completed in December 2019 and the results are still expecting.

IDO-containing MSC-Exos showed beneficial effects in the attenuation of Th17 cell-driven inflammatory diseases, including dry eye disease (DED) [64]. MSC-Exo-based attenuation of DED in patients suffering from chronic GvHD will be examined in a clinical trial that is currently recruiting patients in China (NCT04213248). Total number of 27 patients will receive artificial tears for 2 weeks, followed by MSC-Exos (10 μg/drop, four times a day) for 14 days. Changes in Ocular Surface Disease Index (OSDI) score, Tear break time, Ocular Surface Staining, Best Corrected Visual Acuity (BCVA), Conjunctiva Redness Score (CRS), tear meniscus height and amount of tear secretion will be monitored during the 12 weeks of follow-up.

Therapeutic potential of MSC-Exos in the healing of large and refractory macular holes (MH) in the eyes was the main aim of clinical trial initiated in March 2017 (NCT03437759). Total number of 44 patients should receive 50 or 20 μg MSC-Exos (dissolved in 10 μL of Phosphate-buffered saline). After air-liquid exchange, MSC-Exos will be dripped into vitreous cavity around MH. MSC-Exo-treated patients will be followed up for at least 6 months via BCVA, fundoscopy, optical coherence tomography and physical examination.

A tolerance clinical study on aerosol inhalation of MSC-Exos in healthy volunteers is currently recruiting participants (NCT04313647). According to the study protocol, 10 healthy volunteers (18–45 year old, males and non-pregnant females) will receive one aerosol inhalation of MSCs-Exos (2.0 × 10^8^ nano vesicles/3 mL) and will be followed-up during the next 7 days. In addition to the physical examination and chest imaging, lymphocyte count and concentration of C-reactive protein, D-dimer, IL-1β, IL-2R, IL-6, and IL-8 will be determined. Another clinical trial which will use aerosol inhalation of MSCs-Exos is going to recruit patients in China (NCT04276987). Since animal studies demonstrated that MSC-Exos may promote anti-microbial properties of lung-infiltrated immune cells [65], therapeutic potential of MSC-Exos in alleviation of SARS-CoV-2 infection will be analyzed in this trial. Total number of 30 patients suffering from SARS-CoV-2 infection will receive aerosol inhalation of MSC-Exos (2.0 × 10^8^ nano vesicles/3 mL/daily, for 5 consecutive days). Safety and efficiency of MSC-Exo-based therapy (weaning from mechanical ventilation, improved organ failure, and decreased mortality) will be analyzed during the 28 days of follow-up.

Therapeutic potential of allogeneic MSC-Exos in the treatment of Epidermolysis Bullosa (EB) lesions will be evaluated in the up-coming clinical trial that will recruit 10 patients (NCT04173650). MSC-Exos will be applied topically, once a day for a period of two months. The study will examine a dose limiting toxicity and efficacy of MSC-Exos. The level of healing and scaring of MSC-Exo-treated lesions as well as the change in itching and pain will be bi-weekly assessed during the 60 days of follow-up. The trial will be launched in September 2020 and the estimated study completion date is November 2021.

MSC-Exos may be used as an adjuvant therapy to support and complement other therapeutic modalities. Clinical trial which will investigate MSC-Exos-based prevention and treatment of multiple organ dysfunction syndrome (MODS) in patients who underwent surgical repair of acute type A aortic dissection (SRATAAD) will be conducted in China in up-coming years (NCT04356300). MSC-Exos-based prevention of MODS will be evaluated in 15 patients who will receive MSC-Exos immediately after SRATAAD. Therapeutic potential of MSC-Exos in the treatment of severe MODS (sequential organ failure assessment score (SOFA) ≥10) will be evaluated in another 15 SRATAAD patients who will receive MSC-Exos immediately after the onset of MODS. MSC-Exos will be intravenously injected at a dose of 150 mg, once a day, for 14 days. MODS-related biochemical indexes and SOFA score will be monitored during the 6 months of follow-up.

## 6. Concluding Remarks and Future Perspectives

Results obtained in a large number of preclinical studies strongly suggested therapeutic potential of MSC-Exos in the cell-free treatment of inflammatory and degenerative diseases. Since MSC-Exos had intrinsic homing capabilities similar to their parental cells and were able to protect their cargo from extracellular degradation, these immunoregulatory agents were considered as a new, promising tool for the modulation of local and systemic detrimental immune response. Due to their capacity to deliver genetic material, immunomodulatory proteins, enzymes, and growth factors directly to the recipient cells, MSC-Exos represent an ideal multifunctional delivery system which could modulate intracellular signaling pathways in the specific target cells.

Despite these encouraging findings, it should be noted that there is still a lot of experimental work to be done before MSC-Exos could be widely used in clinical practice. Since a various number of anti-apoptotic and immunosuppressive molecules have been proposed as crucially important for therapeutic effects of MSC-Exos, further experimental studies should identify the exact disease-specific MSC-Exo-contained molecule(s) responsible for enhanced tissue repair and regeneration. Bioengineering and cellular modification techniques should be used to further modify surface of MSC-Exos in other to enhance their ability for cell-specific targeting and, accordingly, to avoid side effects related to the accumulation of MSC-Exos in non-desired cells. Finally, protocols for isolation, storage, and application of MSC-Exos should be optimized for each clinical condition. The exact disease-specific therapeutic dose, the appropriate treatment schedule and route of MSC-Exos administration have to be defined before MSC-Exos will be offered as universal human remedy for the treatment of inflammatory and degenerative diseases.

**Supplementary Paragraph:** An extensive literature review was carried out in March 2020 across several databases (MEDLINE, EMBASE, Google Scholar, and ClinicalTrials.gov), from 1990 to present. Keywords used in the selection were: “mesenchymal stem cells (MSCs)”, “exosomes (Exos)”, “immunomodulation”, “signaling pathways”, “inflammation”, “autoimmune diseases”, “regeneration”, and “therapy”. All journals were considered, and an initial search retrieved 1192 articles. The abstracts of all these articles were subsequently reviewed by two of the authors (C.R.H. and V.V.) to check their relevance to the subject of this manuscript. Eligible experimental and clinical studies had to delineate molecular and cellular mechanisms involved in the beneficial effects of MSC-Exos, and their findings were analyzed in this review.

## Figures and Tables

**Table 1:** Effects of MSC-Exos on immune cells.

Target Cell	Molecular Mechanism	Effect on Cell Phenotype and Function	Therapeutic Potential	Ref.
M1 macrophages	miR-146a-dependent inhibition TRAF6/IRAK1/NF-κB-signaling	Reduced expression of iNOS and inflammatory cytokines (TNF-α, IL-1β, IL-6)	DSS-induced colitis	[15]
HSCs	miR-181-5p-dependent induction of autophagy and apoptosis	Increased expression of Beclin-1 and suppression of Bcl-2	Liver fibrosis	[17]
M2 macrophages and microglial cells	miR-223-5p-dependent expression of Arginase-1	Increased secretion of IL-10 and TGF-β	I/R-induced lung injury; AD and MS	[18,23,24]
DCs	miR-21-based suppression of NF-κB signaling	Reduced expression of co-stimulatory molecules (CD40, CD80, CD86), decreased production of Th1 and Th17-related cytokines (IL-1β, IL-6, and IL-12)	EAU; EAE; STZ-T1DM	[28,29,30]
Th1 and Th17 cells	TGF-β-dependent inhibition of JAK/STAT signaling	G1 cell cycle arrest and reduced proliferation	EAE; STZ-T1DM	[29,30]
Tregs	TGF-β and IDO-dependent activation of GCN2 kinase	Reduced transdifferentiation of Tregs in Th17 cells	STZ-T1DM; chronic airway inflammation	[30,40]
B cells	miR-dependent down-regulation of JCHAIN, PTGS2, POU2AF1, TNFRSF13B, SH2D1A, LTA genes	Suppressed proliferation and reduced production of IgM.	B cell-mediated autoimmune diseases	[42]

Abbreviations: TNF receptor-associated factor 6 (TRAF6); IL-1 receptor-associated kinase 1 (IRAK1); inducible nitric oxide synthase (iNOS); tumor necrosis factor alpha (TNF-α); dextran sulfate sodium (DSS); hepatic stellate cells (HSCs); transforming growth factor beta (TGF-β); ischemia/reperfusion (I/R); Alzheimer’s disease (AD); multiple sclerosis (MS); dendritic cells (DCs); experimental autoimmune uveoretinitis (EAU); experimental autoimmune encephalomyelitis (EAE); streptozotocin-induced model of type-1 diabetes mellitus (STZ-T1DM); indoleamine-2,3-dioxygenase (IDO); indoleamine 2,3 dioxygenase; general control nonderepressible 2 (GCN2) kinase.
